# Improved Glycemic Control With a Digital Health Intervention in Adults With Type 2 Diabetes: Retrospective Study

**DOI:** 10.2196/28033

**Published:** 2021-06-02

**Authors:** Gretchen Zimmermann, Aarathi Venkatesan, Kelly Rawlings, Michael D Scahill

**Affiliations:** 1 Vida Health San Francisco, CA United States

**Keywords:** type 2 diabetes, digital health, diabetes intervention, diabetes, mobile health, mHealth, app-based, health coaching, HbA1c, glycemic improvements

## Abstract

**Background:**

Traditional lifestyle interventions have shown limited success in improving diabetes-related outcomes. Digital interventions with continuously available support and personalized educational content may offer unique advantages for self-management and glycemic control.

**Objective:**

In this study, we evaluated changes in glycemic control among participants with type 2 diabetes who enrolled in a digital diabetes management program.

**Methods:**

The study employed a single-arm, retrospective design. A total of 950 participants with a hemoglobin A_1c_ (HbA_1c_) baseline value of at least 7.0% enrolled in the Vida Health Diabetes Management Program. The intervention included one-to-one remote sessions with a Vida provider and structured lessons and tools related to diabetes management. HbA_1c_ was the primary outcome measure. Of the 950 participants, 258 (27.2%) had a follow-up HbA_1c_ completed at least 90 days from program start. Paired *t* tests were used to evaluate changes in HbA_1c_ between baseline and follow-up. Additionally, a cluster-robust multiple regression analysis was employed to evaluate the relationship between high and low program usage and HbA_1c_ change. A repeated measures analysis of variance was used to evaluate the difference in HbA_1c_ as a function of the measurement period (ie, pre-Vida enrollment, baseline, and postenrollment follow-up).

**Results:**

We observed a significant reduction in HbA_1c_ of –0.81 points between baseline (mean 8.68, SD 1.7) and follow-up (mean 7.88, SD 1.46; *t*_257_=7.71; *P*<.001). Among participants considered high risk (baseline HbA_1c_≥8), there was an average reduction of –1.44 points between baseline (mean 9.73, SD 1.68) and follow-up (mean 8.29, SD 1.64; *t*_139_=9.14; *P*<.001). Additionally, average follow-up HbA_1c_ (mean 7.82, SD 1.41) was significantly lower than pre-enrollment HbA_1c_ (mean 8.12, SD 1.46; *F*_2, 210_=22.90; *P*<.001) There was also significant effect of program usage on HbA_1c_ change (β=–.60; *P*<.001) such that high usage was associated with a greater decrease in HbA_1c_ (mean –1.02, SD 1.60) compared to low usage (mean –.61, SD 1.72).

**Conclusions:**

The present study revealed clinically meaningful improvements in glycemic control among participants enrolled in a digital diabetes management intervention. Higher program usage was associated with greater improvements in HbA_1c_. The findings of the present study suggest that a digital health intervention may represent an accessible, scalable, and effective solution to diabetes management and improved HbA_1c_. The study was limited by a nonrandomized, observational design and limited postenrollment follow-up data.

## Introduction

Diabetes continues to plague the United States and the rest of the globe [1]. An estimated 34.1 million adults, 13% of the US adult population, have diabetes, with just under 80% diagnosed [2]. Amid the seemingly inexorable rise, it can be easy to forget what a truly modern phenomenon this is. In *The Principles and Practice of Medicine* of 1892, William Osler estimated a diabetes prevalence of just 2.8 per 100,000 in the United Sates, which, in his day, lumped together both types 1 and 2 [3]. This modernity would seem to suggest the tide can be rolled back if only its causes were understood, but, alas, the disease marches on [4].

Among those with diabetes, disease control is clearly a major challenge. Although clinical guidelines broadly agree on hemoglobin A_1c_ (HbA_1c_) targets of 7.0% or less for most people with diabetes, some 50% of those diagnosed are, by this standard, not on target, and thus at elevated risk of macrovascular and microvascular complications [2,5,6]. This serves only to highlight the challenges people with diabetes face. It is a disease that requires daily attention to and navigation of myriad decisions—choosing foods, taking medication, monitoring blood glucose, and accessing preventive and acute care [7]. Although diabetes self-care behaviors have been found to be positively correlated with improved glycemic control and quality of life, clearly many people with diabetes struggle to adopt such behaviors [8].

With great prevalence and barriers to control comes great cost. In 2017, total estimated costs of diabetes in the United States were US $327 billion, of which US $237 billion came from direct medical costs [9]. A safe, effective, efficient, and scalable intervention would be welcome. Many drug trials have shown disappointing results notably with no improvement in macrovascular outcomes in the UK Prospective Diabetes Study (UKPDS) 33 trial and increased mortality despite lower HbA_1c_ achieved in the Action to Control Cardiovascular Risk in Diabetes (ACCORD) trial [10,11]. Lifestyle interventions have similarly seen prominent disappointments in the Look AHEAD and MOVE! projects [12,13]. Some interventions, such as the Diabetes Remission Clinical Trial (DiRECT), have shown promise, but it remains unclear whether strategies that include such intensive interventions as meal replacement can be scaled up to the millions of people living with diabetes in highly varied social, economic, and cultural settings [14-16].

Digital health may offer some solutions. Traditional outpatient interventions, however extensive, are limited by their sporadic nature and thus leave a substantial burden on the patient to internalize behaviors. A digital solution has the potential to deliver guidance and support anywhere and anytime it may be needed. Preliminary efforts to this effect have shown promise to the point that diabetes care standards already recognize the potential benefits of “connected care” [17-21].

Benefits may include increased access to care and health improvements. In addition to removing traditional barriers to face-to-face interactions, such as transportation and daytime office hours, digital platforms are linked to mental and metabolic outcomes. Small randomized controlled trials of these programs have found improvements in diabetes self-care behaviors and self-efficacy along with glycemic and mental health measures [22,23]. A common theme in qualitative analyses of these interventions is the perception of feeling connected at all times to a human who cares [23].

Operationalizing the effect of a “human who cares” via a digital platform does come with challenges. As Markert et al [24] note in a literature review of telehealth coaching for seniors, it can be challenging and time consuming to foster a therapeutic relationship and tailor the intervention to the individual.

Furthermore, there is little standardization of digital intervention components in both the literature and products in the market. Greenwood et al [25] conducted a systematic review of technology-enabled diabetes management interventions. Of these interventions, 18 reported significant reductions in HbA_1c_ albeit with heterogeneity in intervention components and methodologies. They did identify 4 key intervention elements present with HbA_1c_ reduction: two-way communication, patient-generated health data tracking or analysis, education, and feedback. These elements are cornerstones of the Vida Health program.

Vida Health is an app-based digital health platform for chronic disease prevention and management. Vida Health is available as an employee benefit through select health plans and direct to consumers across the United States. Type 2 diabetes management is one of the core offerings on the Vida Health platform. Vida’s platform combines mobile technology and human-centered digital coaching to foster shared decision-making, goal setting, and accountability between provider and patient in daily diabetes self-care. App content covers a wide spectrum of lifestyle priorities including nutrition, blood glucose self-monitoring, and medication management. From a standard initial sequence, content is rapidly tailored to patient needs using both machine-learning recommendation algorithms and provider input. Our hypothesis was that this continuously available, highly personalized combination of provider guidance and content would drive improvements in diabetes control as assessed by changes in HbA_1c_. We further hypothesized that app-based usage would be positively correlated with HbA_1c_ improvements.

## Methods

### Design and Measures

A single-arm, retrospective design was used to investigate the impact on HbA_1c_ in adults whose baseline value reflected suboptimal type 2 diabetes control and who enrolled in Vida Health’s app-based digital health intervention. The study was approved by an independent institutional review board (Western Institutional Review Board, Inc), which waived informed consent because the study was identified as having minimal risk and because the data were fully anonymized before use in the analysis.

### Study Sample and Recruitment

The study included adults (18 years or older) from 2 major insurance carriers that were clients of Vida Health, and so participants received the Vida Health Program free of charge. HbA_1c_ data were obtained directly from these insurance carriers via their data sharing arrangements with outpatient laboratory networks. Participants were eligible for the study if they had a baseline HbA_1c_ value of at least 7.0% (as described in Statistical Analysis). All participants in the study were enrolled in the Vida Health Diabetes Management Program (henceforth “Program”), had smartphone or web-based access, and were fluent in spoken and written English or Spanish. Vida has made the Program available in both English and Spanish through professional translation and employs bilingual providers.

Eligible participants were recruited through a combination of brochures, outbound calling campaigns, and email announcements with general information provided about the Program and how to enroll. They were directed to download the Vida Health app from the Apple App Store (Apple Inc) or Google Play Store (Google) and to enter an invitation code to confirm insurance coverage.

After installing the app and prior to enrolling in the Vida Health Program, participants were presented with a series of brief in-app intake forms through which they provided contact information, basic demographic information (self-reported weight, height, age, and gender), and existing health conditions. Informed consent for digital nutrition therapy was a standard part of the initial app content. Exclusion criteria were type 1 diabetes, chronic kidney disease stages 4 or 5, congestive heart failure classes III or IV, pregnancy, and breastfeeding.

### Therapeutic Approach and Intervention

The Program is a digital diabetes intervention program with remote coaching sessions encouraged up to weekly for the first 12 weeks and monthly thereafter. Participants are paired with a Vida provider—certified health coach, registered dietitian, or certified diabetes care and education specialist—who specializes in diabetes self-management. Vida providers receive intensive evidence-based training on motivational interviewing techniques that promote self-efficacy and autonomy for behavior change [26].

The Program combined one-to-one support, educational content, biomarker tracking, and data analysis to address self-care behaviors. Provider support was delivered through live in-app audio-video sessions (audio-only also available) and text messaging. The initial encounter included a detailed health assessment. The Vida provider used motivational interviewing to guide the participant in defining the initial area of focus for lifestyle change and identifying any associated barriers. Subsequent sessions followed up on these goals and worked to resolve ambivalence to change. Each session concluded with an individualized wellness plan including specific goals. Between counseling sessions, participants were encouraged to text message their Vida provider for further support. The Vida provider used text messaging to offer feedback on data tracking and motivational interviewing to overcome barriers to change.

**Figure 1:**
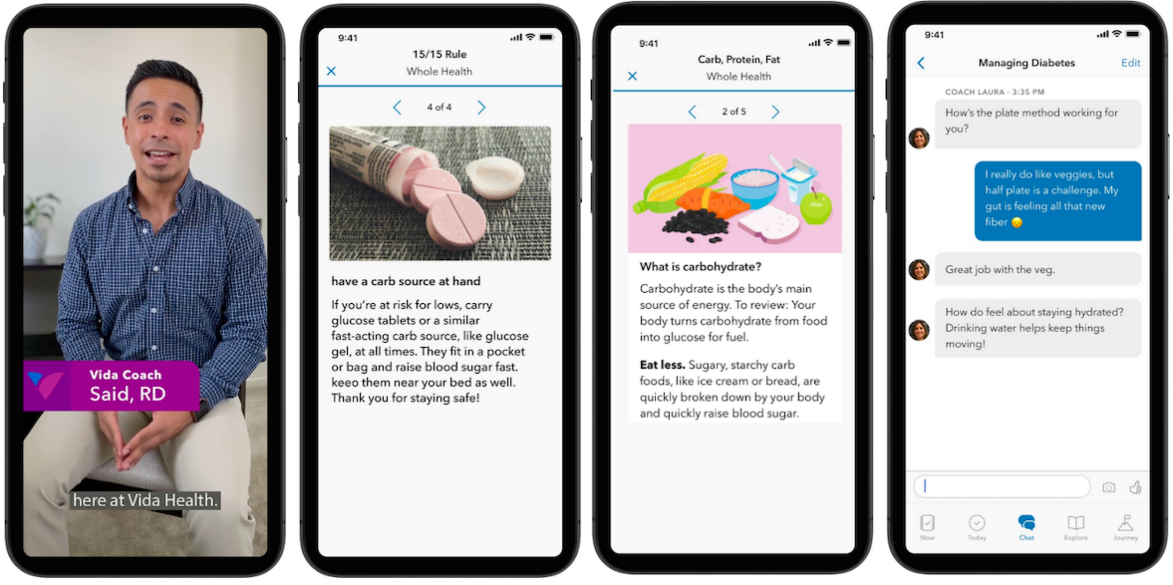
Vida Health educational content screens.

App content was the primary emphasis to support scalability. It included structured lessons and multimedia content (see Figure 1) with evidence-based approaches to health behavior change, such as blood glucose self-monitoring, medication adherence, and nutrition [27]. Participants could review and interact with the lessons by responding to question prompts therein. The Vida provider reviewed completed lessons to help members apply their learnings to their goals and diabetes self-management behaviors.

For those participants who reported having been recommended self-monitoring of blood glucose, logging was encouraged. The Vida app supports connections to a variety of commercially available cellular connected blood glucose meters and also allows for manual logging of data. Structured logging capabilities for food intake and physical activity are also available.

### Statistical Analysis

The primary outcome measure for this study was HbA_1c_. HbA_1c_ was measured in clinical laboratories and the data made available by Vida Health’s payer clients. Baseline HbA_1c_ was defined as the laboratory test closest to Program start, measured between 6 months before to within 21 days after enrollment. For 7 participants, HbA_1c_ laboratory data results were >14. In these instances, we confirmed the Logical Observation Identifiers Names and Codes (LOINC) test description as HbA_1c_ and the result unit of measurement as % of total hemoglobin and used a conservative approach to assign a HbA_1c_ value of 14.0.

The follow-up measure was defined as a HbA_1c_ test completed a minimum of 90 days post Program start. In order to evaluate possible systematic baseline differences between participants with a valid follow-up measure and those with no follow-up, we performed a 2-tailed chi-square test to assess gender-based differences. Additionally, a set of 2-tailed *t* tests were employed to evaluate differences between groups based on age and baseline HbA_1c_.

A paired *t* test was used to assess change in HbA_1c_ from baseline. A repeated measures analysis of variance (ANOVA) with the measurement period as a within-subject factor was used to analyze changes in HbA_1c_ from the pre-enrollment measure to baseline and from baseline to follow-up. Pre-enrollment was defined as a HbA_1c_ measure obtained at least 90 days prior to the baseline. A Mauchly test was used to confirm that assumptions of sphericity had not been violated. We conducted a series of post hoc pairwise comparisons of means to evaluate HbA_1c_ changes between each measurement window.

Program usage was a secondary focus of this study. Although conceptually related to user engagement, program usage or adherence comprises objective measures of a user’s interaction with the digital interface over time (eg, number of log-ins, counseling sessions, lessons and tools completed). User engagement, on the other hand, includes the subjective experience of the digital intervention with a focus on the quality of the experience [28,29]. Although the behavioral aspect of engagement (usage) and the subjective or experiential aspect (eg, satisfaction, interest, perceived relevance) can no doubt influence one another, their independent or interactive effect on clinical outcomes in the context of digital health remains unclear [29]. Measures of the experiential dimension of engagement were not assessed in this study. Program usage was conceptualized using 3 in-app behaviors. First, we computed a cumulative sum for each of the following factors: number of counseling sessions, number of messages sent to the provider by the participant, and the number of lessons completed within the first 6 weeks of Program start. We then created a binary program usage variable where high usage was defined as participants with greater-than-or-equal-to-median coach interaction and greater-than-or-equal-to-median content interaction. A cluster-robust multiple regression analysis was used to evaluate the association between the extent of usage and HbA_1c_ change. Data preparation and analyses were performed using Python Version 3.7.7 and Stata/IC 16.0 (StataCorp).

### Data Availability

The data sets analyzed for this study are available from the corresponding author upon request.

## Results

### Member Characteristics

**Figure 2:**
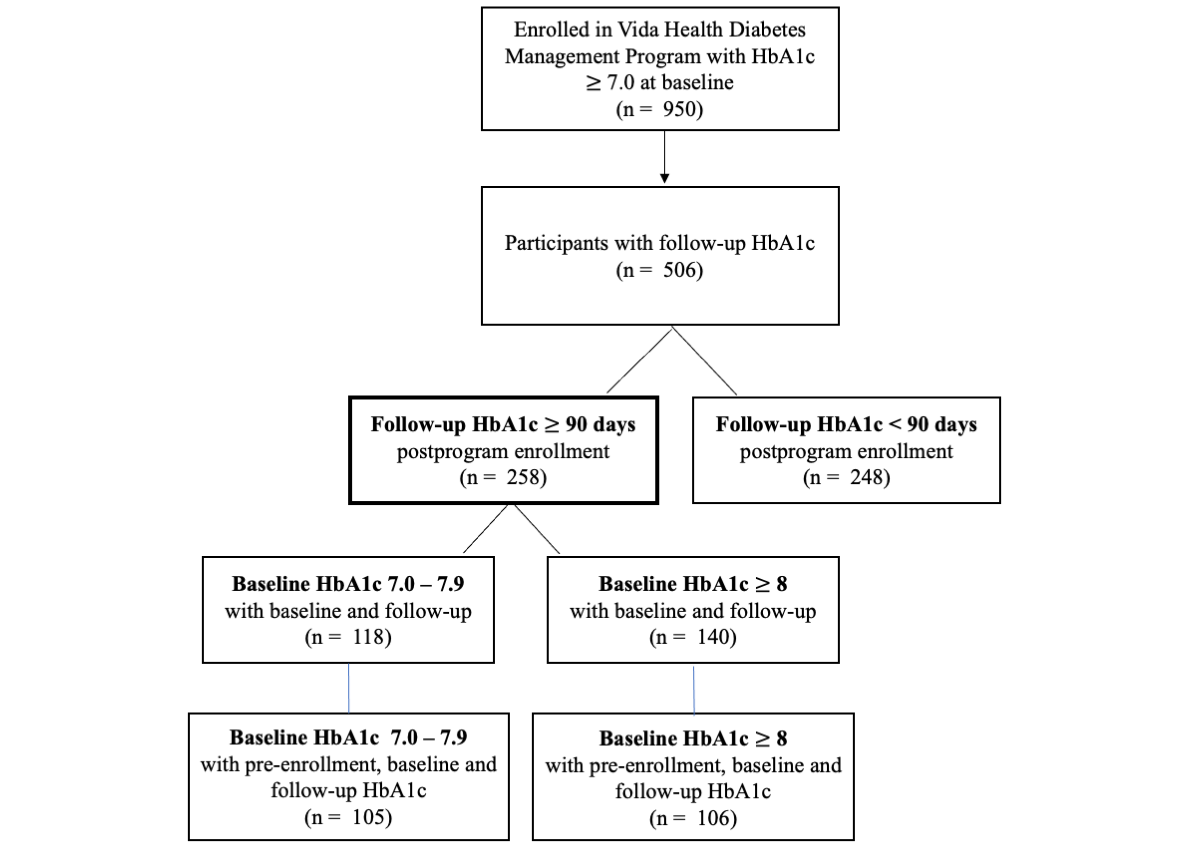
Schematic of participant flow. HbA_1c_: hemoglobin A_1c_.

In all, 950 participants enrolled in the Vida Health Diabetes Management Program. A total of 692 participants (72.8%) had no postenrollment follow-up HbA_1c_ value, which was defined as a HbA_1c_ test completed at least 90 days from Program start. A schematic of participant flow is presented in Figure 2. Of the 692 participants with no follow-up, 248 (35.5%) did have a follow-up HbA_1c_ laboratory measure available; however, the measure was completed within 90 days of program start. Because our a priori definition of follow-up, based on the physiological characteristics of the HbA_1c_ test, was a laboratory test obtained a minimum of 90 days after Program start, participants with a postenrollment test obtained before 90 days were excluded from the outcome analyses and were considered to be missing a 3-month follow-up measure [30]. Basic demographics of the study cohort are presented in Table 1.

A 2-tailed *t* test revealed no significant group-level differences in baseline HbA_1c_ levels between follow-up HbA_1c_ availability status (*t*_948_=1.27; *P*=.21). Participants with a valid follow-up HbA_1c_ appeared to be younger (mean 56.79, SD 9.52) than those without a follow-up available (mean 60.22, SD 11.8; *t*_948_=4.18; *P*<.001). Additionally, a 2-tailed chi-squared test showed no significant gender-based differences between the groups (*X* ^2^=0.46; *P*=.79). Average baseline HbA_1c_ for the study cohort was 8.79 (SD 1.62).

**Table 1:** Demographic characteristics of the study cohort (N=950).

Group			Count (n)		Proportion (%)		Age (years), mean (SD)		Baseline HbA_1c_^a^, mean (SD)
**No post-90 day HbA_1c_ follow-up available (n=692)**			692		72.8		60.22 (11.80)		8.83 (1.59)
	Female	405		42.6		59.72 (10.98)		8.83 (1.66)	
	Male, n (%)	286		30.1		60.91 (12.87)		8.84 (1.48)	
	Unspecified	1		0.1		63.00^b^		7.10^b^	
**Post-90 day HbA_1c_ follow-up available (n=258)**			258		27.2		56.79 (9.52)		8.68 (1.70)
	Female	154		16.2		55.85 (9.38)		8.74 (1.76)	
	Male	104		11.0		58.19 (9.60)		8.60 (1.62)	
Overall			950		100		59.29 (11.32)		8.79 (1.62)

^a^HbA_1c_: hemoglobin A_1c_.

^b^SD value is not applicable.

### Primary Outcome

**Figure 3:**
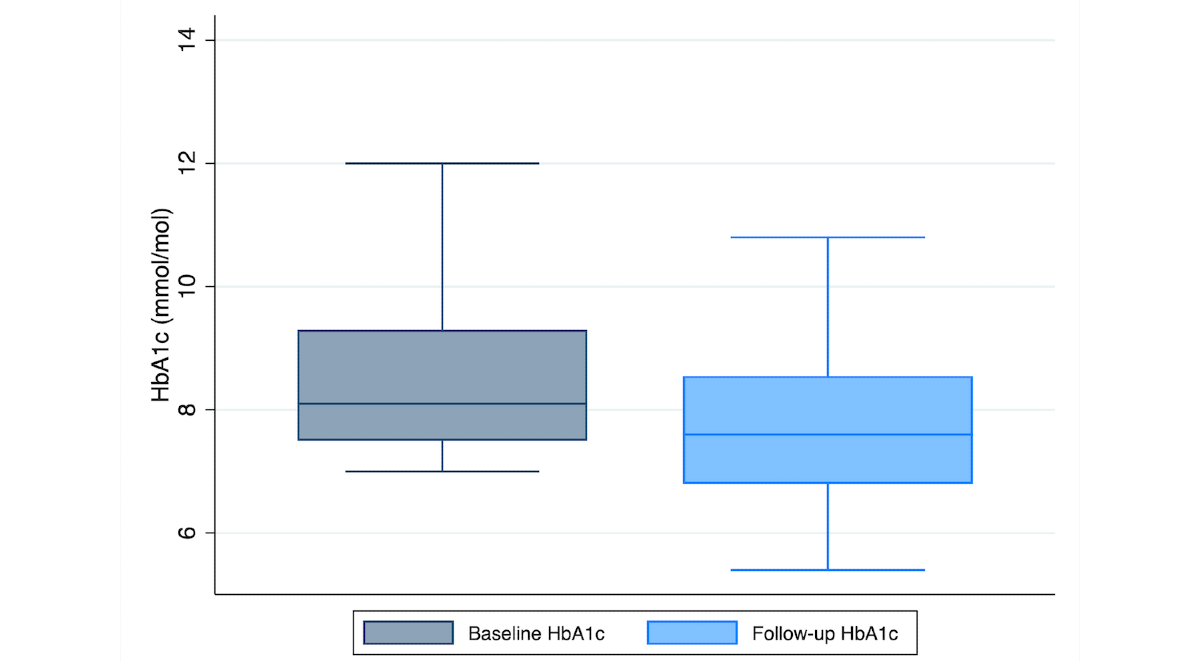
A boxplot of HbA_1c_ at baseline and a minimum 90-day follow-up (N=258). HbA_1c_: hemoglobin A_1c_.

**Figure 4:**
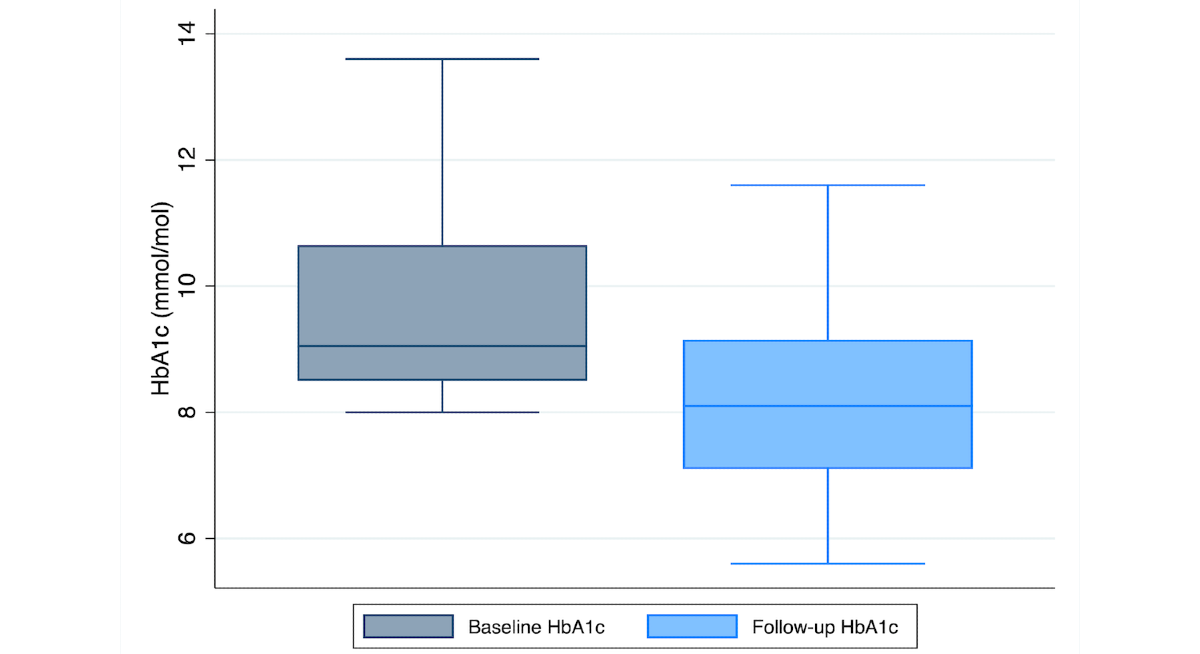
A boxplot of change in HbA_1c_ from baseline to follow-up among high-risk participants with a baseline HbA_1c_ ≥ 8 (N=140). HbA_1c_: hemoglobin A_1c_.

Follow-up HbA_1c_ measurements were completed on average 132.68 days (SD 31.46) from Program start. As shown in Figure 3, a paired *t* test revealed a significant reduction in HbA_1c_ of –0.81 points between baseline (mean 8.68, SD 1.7) and follow-up (mean 7.88, SD 1.46; *t*_257_=7.71; *P*<.001). Among high-risk participants with a baseline HbA_1c_ ≥8, we observed an average reduction of –1.44 points between baseline (mean 9.73, SD 1.68) and follow-up (mean 8.29, SD 1.64; *t*_139_=9.14; *P*<.001; see Figure 4).

In terms of provider interaction, a majority (167/258, 64.7%) had completed at least one provider session within the first 6 weeks of the program. Although both groups did have a significant decrease in HbA_1c_ relative to follow-up, change in HbA_1c_ varied as a function of consultation status (*t*=2.63; *P*<.001) such that participants who had completed at least one counseling session with a provider had a greater decrease in HbA_1c_ (mean –1.00, SD 1.66) compared to those who had never completed a session (mean –0.44, SD 1.65). A similar pattern emerged for the high-risk cohort (baseline HbA_1c_ ≥8). Among participants with baseline HbA_1c_ ≥8, the majority (93/140, 66.4%) had completed at least one counseling session within the first 6 weeks of program start. A Welch *t* test revealed a significant difference in HbA_1c_ change as a function of session status, (*t*=2.34; *P*=.02). Participants who had completed at least one session had an average reduction of –1.71 points (SD 1.70) compared to participants who had yet to complete a session (mean –0.90, SD 2.04).

**Figure 5:**
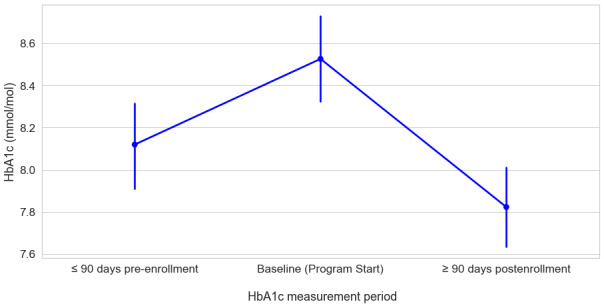
Estimated marginal means of HbA_1c_ as a function of measurement period (N=211). HbA_1c_: hemoglobin A_1c_.

In order to evaluate the effects of no program intervention, we used a repeated measures approach. The model included HbA_1c_ measured at 3 time points (time 1: HbA_1c_ from at least 90 days up to 12 months prior to the baseline HbA_1c_ test; time 2: baseline HbA_1c_; time 3: minimum 90-day postenrollment HbA_1c_ follow-up). The vast majority of participants (211/258, 81.7%) had data available for all 3 time points. A 1-way repeated measures ANOVA was conducted to assess the effect of measurement time point on HbA_1c_ prior to, at baseline, and at follow-up. A Mauchly test of sphericity confirmed that the assumption of sphericity had not been violated (*X*^2^=5.91; *P*=.05). We observed a significant effect of measurement time on HbA_1c_, (*F*_2, 210_=22.90; *P*<.001). A post hoc pairwise comparison of marginal means between measurement time points showed a significant increase between pre-enrollment HbA_1c_ (mean 8.12, SD 1.46) and baseline (mean 8.53, SD 1.56; *t*_210_=3.90; *P*<.001). As anticipated, we observed a significant decrease from baseline to 90-day postenrollment follow-up (mean 7.82, SD 1.41; *t*_210_=–6.74; *P*<.001). Additionally, we noted that follow-up HbA_1c_ (mean 7.82, SD 1.41) was significantly lower than pre-enrollment HbA_1c_, (mean 8.12, SD 1.46; *t*_210_=–2.84; *P*=.005. In summary, we observed an increase in HbA_1c_ between pre-enrollment and baseline but a significant reduction between baseline and follow-up (see Figure 5).

**Figure 6:**
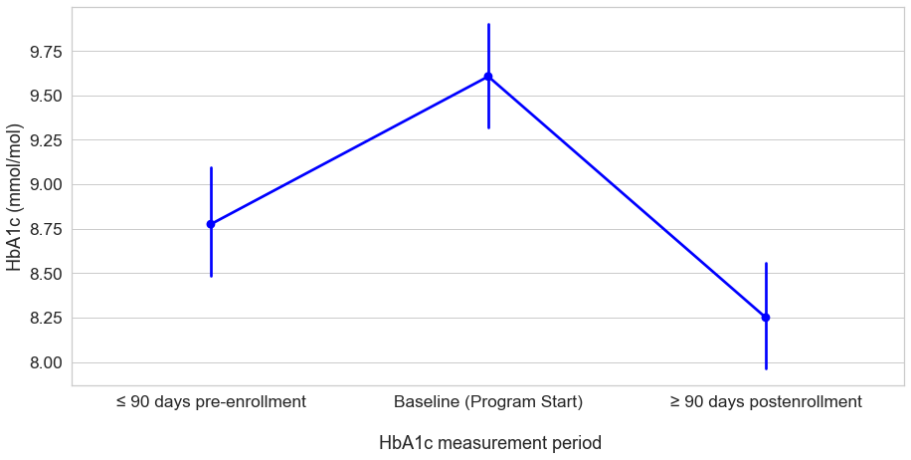
Changes in HbA_1c_ as a function of measurement period among high-risk participants with a baseline HbA_1c_ ≥8 (N=106).

A similar pattern of results emerged among participants with a HbA_1c_ ≥8. In this high-risk cohort, 75.7% (106/140) of the participants had data available for the 3 measurement periods. As shown in Figure 6, there was a significant effect of measurement period HbA_1c_, (*F*_2,105_=31.6; *P*<.001). A post hoc pairwise comparison of marginal means revealed a significant increase between pre-enrollment HbA_1c_ (mean 8.78, SD 1.57) and baseline (mean 9.60, SD 1.56; *t*_105_=0.83; *P*<.001). As expected, there was a significant decrease in HbA_1c_ from baseline to follow-up (mean 8.25, SD 1.61; *t*_105_=–7.88; *P*<.001). Additionally, we noted the 90-day postenrollment HbA_1c_ (mean 8.25, SD 1.61) was significantly lower than the average pre-enrollment HbA_1c_ (mean 8.78, SD 1.57; *t*_105_=–0.52; *P*=.003).

### Program Usage Outcomes

We hypothesized that program usage would be associated with improvements in HbA_1c_ reduction at follow-up. In order to test this hypothesis, we used a cluster-robust linear regression model that included all participants with postenrollment follow-up HbA_1c_ data, irrespective of days between baseline and follow-up. Active program usage was operationalized as a binary variable that was derived from 3 measures of program usage within the first 6 weeks of enrollment: number of sessions, number of messages sent to the provider, and number of lessons completed. These factors were left skewed, indicative of possible “super users” of the app (see Table 2). Therefore, we used the median to define cutoffs for high and low program usage. High usage was defined as participants having completed at least 2 sessions or having sent at least 7 messages to their provider and completed at least 4 lessons in the app within the first 6 weeks of the program. These cutoffs were determined using the median value for each of these factors. Greater relative usage of lessons and app content relative to provider sessions was expected given Program design. A 2-tailed *t* test revealed no significant difference in baseline HbA_1c_ between the high and low program usage groups (*P*=.25). Based on the above described cutoffs, 47.2% (122/258) of participants were considered to have high usage, and 52.7% (136/258) were considered to have low usage.

**Table 2:** Summary statistics for Program usage within the first 6 weeks of the Program (N=258).

Statistic	Number of sessions	Number of messages	Number of in-app lessons
Mean	2.8	17.30	8.17
SD	2.61	26.53	9.72
Median	2	7	3.5

**Figure 7:**
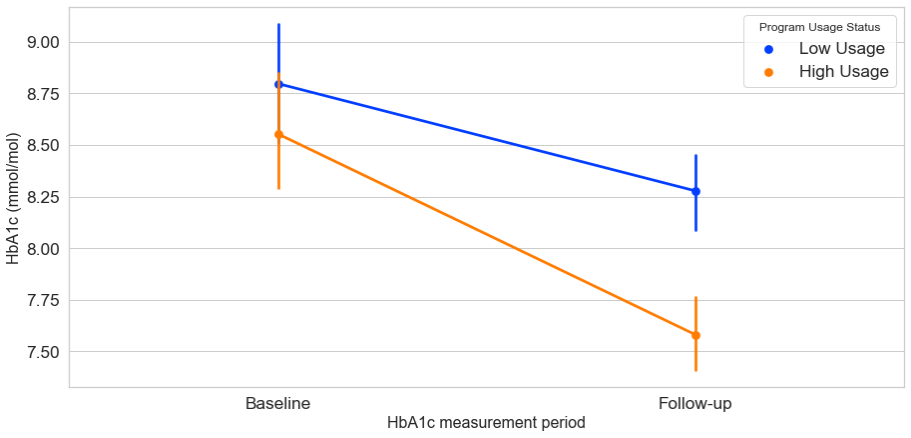
Estimated marginal means of HbA_1c_ at Program start (baseline) and at follow-up as a function of high and low program usage (N=258).

Gender, age, time to follow-up, and the binary program usage variable were included as fixed factors and baseline HbA_1c_ as a covariate. We employed a cluster-robust multiple regression analysis to account for possible differences in provider effectiveness. Change in HbA_1c_ was defined as the outcome variable. As shown in Figure 7, we observed a significant main effect of usage on change in HbA_1c_, (β=–.60; *P*<.001), such that high usage was associated with a greater decrease in HbA_1c_ at follow-up (M_high-usage_=–1.02; SD_high-usage_=1.60; mean_low-usage=_–.61; SD_low-usage=_1.72). A higher baseline HbA_1c_ was associated with greater improvement in HbA_1c_ (β=–.63; *P*<.001) such that participants with a higher baseline HbA_1c_ showed a greater decrease at follow-up. We observed no significant effect of time to follow-up (measured in days), age, or gender.

## Discussion

### Principal Findings

The objective of this study was to assess the effectiveness of Vida’s digitally delivered continuous care platform on HbA_1c_ improvement. In this retrospective study of 258 participants with suboptimally controlled type 2 diabetes (baseline HbA_1c_ ≥7) who enrolled in an app-based digital health intervention paired with one-to-one remote health coaching, we observed an average reduction in HbA_1c_ of –0.81 at post-90 day follow-up relative to baseline. Among participants considered high-risk (baseline HbA_1c_ ≥8), we observed a stronger average reduction in HbA_1c_ (mean –1.44 points, SD 1.86) relative to baseline. We used a repeated measures approach in which participants serve as their own control to evaluate changes in HbA_1c_ pre- and postenrollment in the Vida Health Program. As detailed below, a substantial portion of the initial cohort has not yet obtained a follow-up HbA_1c_. It is, however, notable that follow-up HbA_1c_ was significantly lower even than the average pre-enrollment HbA_1c_.

The results also provide preliminary insight into the role of program usage as a possible moderator of glycemic control. The majority of the study cohort had completed at least one session with their provider within the first 6 weeks of the Program. Both groups showed a significant decrease in HbA_1c_, while participants who had at least one session showed greater improvement in HbA_1c_ (mean –1.00, SD 1.66) than those who had yet to complete a session. A similar pattern emerged when we operationalized usage in the digital platform as a combination of provider-reliant actions (ie, video call sessions and asynchronous messaging) and app-based interactions (eg, reading lessons, viewing multimedia content). We observed a significant positive association between program usage and improved glycemic control. The analysis revealed a statistically significant and clinically meaningful reduction in HbA_1c_ from baseline to follow-up at least 3 months postenrollment. A growing body of evidence suggests digital interventions as an innovative way to deliver and engage people in their diabetes care. Yet, many open questions remain about the best combination of provider interaction and other components, such as educational content, interactive prompts, and data tracking, via a digital platform. The Vida hybrid model of digital diabetes management suggests an effective, scalable way to improve key diabetes-related health outcomes.

Although the Vida experience, including content and data tracking, can be navigated without provider contact and in a self-paced manner, we observed that the combination of human interaction and content app components is associated with improved HbA_1c_. In a secondary outcome measures analysis, we observed a significant effect of usage, as defined by interaction with the provider and the digital platform. Higher program usage was associated with a more pronounced reduction of HbA_1c_. This suggests Vida’s hybrid model, which offers a combination of curated and self-paced app-based components and ongoing support from a human provider, may offer advantages over a hypothetical analogue without a human provider. Although the study design lacked a control group or matched comparison group, and so prevents causal inference, it suggests promising avenues for future research. A flexible, scalable solution to population-level diabetes management would certainly be welcome.

The provider interaction that shows correlation with outcomes here provides a vehicle for motivational interviewing throughout a participant’s experience. Motivational interviewing has been shown to be effective in supporting diabetes self-care [26,31,32]. Vida providers receive extensive training and ongoing evaluation in behavioral counseling with motivational interviewing techniques being a core component. Further research is warranted to explore if this human interaction and motivational interviewing methodology explain these atypical improvements in HbA_1c_.

### Limitations

This study used a nonrandomized, observational design that does not allow for causal inferences about the intervention and its impact on the primary outcome measure, change in HbA_1c_. Participants self-selected to enroll in Vida and so may be more motivated to change their behavior and improve their health than those who were eligible but did not enroll. Similarly, while eligible participants were not known by their insurance carrier to be engaged with a similar diabetes intervention, simultaneous efforts to explain the effect cannot be excluded. This may be a particular concern among those with lower usage. Furthermore, while the program was completely free at the point of care to participants, the participant sample included only individuals with health insurance and so does not well represent underserved populations.

Despite possible systematic baseline differences between groups based on age—HbA_1c_ at baseline and gender—no significant gender-based differences were observed. Age-based differences were observed only for the presence of valid HbA_1c_ follow-up data. Of the 950 participants who enrolled, a total of 692 participants (72.8%) did not have a postenrollment follow-up HbA_1c_ value, defined as a HbA_1c_ test completed at least 90 days from Program start. Of the 692 participants with no follow-up, 248 (35.5%) did have a follow-up HbA_1c_ laboratory measure available; however, the measure was completed within 90 days of Program start, and thus this cohort was not included in the outcomes analysis due to the clinical significance of these values being difficult to interpret. The lack of follow-up HbA_1c_ among these participants is likely multifactorial. Participants with valid HbA_1c_ follow-up data were younger than those without a follow-up. Given the fact that the majority of the participants were enrolled in this study during the COVID-19 pandemic, it is reasonable to assume that access to HbA_1c_ tests might have been impacted by stay-at-home orders, restrictions on nonemergent care, and public health communication about COVID-19 risks and that these might have particularly impacted older participants [33]. With these limitations related to participants, the findings are not generalizable to all adults with type 2 diabetes.

Limitations with the secondary outcome include the modest sample size and the related numbers of provider and content interactions that stratified program usage. It may be that with a different definition of program usage or engagement, we would see a different impact on HbA_1c_ outcomes. Acceptability of the intervention for participants cannot be assessed by the study or generalized to other people living with diabetes.

Finally, the analysis examined only the intensive, first 12-week portion of the Vida Program, which is offered to participants as a 1-year experience. No inferences are possible about the persistence of usage with the digital health intervention or of sustained HbA_1c_ improvements until further follow-up data become available.

### Conclusions

In this study, adults with type 2 diabetes were enrolled in the Vida Health digital diabetes management program with rich educational content and one-to-one coaching grounded in motivational interviewing. The results of this study indicate statistically significant and clinically meaningful glycemic improvements post intervention. The nonrandomized observational design, modest sample size, and low number of participants who met the follow-up HbA_1c_ criteria were study limitations. Although further research will be welcome, evidence-based, digitally delivered interventions like Vida Health may already represent an accessible, scalable, and effective solution to diabetes management and improved HbA_1c_.

## Abbreviations

ACCORD: Action to Control Cardiovascular Risk in Diabetes
ANOVA: analysis of variance
DiRECT: Diabetes Remission Clinical Trial
HbA_1c_: hemoglobin A_1c_
LOINC: Logical Observation Identifiers Names and Codes
UKPDS: UK Prospective Diabetes Study
